# Electromyography After Total Hip Arthroplasty: A Systematic Review of Neuromuscular Alterations and Functional Movement Patterns

**DOI:** 10.3390/jcm15010400

**Published:** 2026-01-05

**Authors:** Maria Cesarina May, Andrea Zanirato, Luca Puce, Eugenio Giannarelli, Carlo Trompetto, Lucio Marinelli, Matteo Formica

**Affiliations:** 1 IRCCS Ospedale Policlinico San Martino, 16132 Genoa, Italy; m_may_93@web.de (M.C.M.); eugeniogiannarelli@gmail.com (E.G.); ctrompetto@neurologia.unige.it (C.T.); lucio.marinelli@unige.it (L.M.); matteo.formica@unige.it (M.F.); 2 Department of Integrated Surgical Diagnostic Sciences (DISC), Orthopedic Clinic, University of Genoa, 16132 Genoa, Italy; 3 Department of Neuroscience, Rehabilitation, Ophthalmology, Genetics, Maternal and Child Health (DINOGMI), University of Genoa, 16132 Genoa, Italy

**Keywords:** neuromuscular dysfunction, hip joint stability, post-arthroplasty motor control, lower-limb muscle coordination, functional recovery assessment, gait analysis, kinematic evaluation

## Abstract

**Background**: Electromyography (EMG) is increasingly used to characterize neuromuscular alterations after total hip arthroplasty (THA), yet available evidence remains fragmented and inconsistent. This systematic review synthesizes postoperative EMG findings during gait, functional tasks, and static assessments, highlighting clinical implications and future research needs. **Methods**: Peer-reviewed studies employing surface, needle, or high-density EMG after THA were systematically examined. Extracted variables included activation amplitude, timing (onset, offset, burst duration), co-activation patterns, and the influence of surgical approach. Methodological rigor, normalization procedures, and the extractability of quantitative EMG metrics were also assessed. **Results**: Across studies, postoperative EMG consistently revealed non-physiological activation patterns, including delayed or prolonged gluteus medius activity and excessive recruitment of posterior chain muscles. These abnormalities persisted for up to 12 months and, in isolated cases, beyond a decade. Comparisons of surgical approaches demonstrated early denervation signs and impaired recruitment following lateral-based incisions, whereas later adaptations differed between lateral and posterior approaches but remained abnormal in both. Needle EMG studies confirmed transient involvement of muscles innervated by the superior gluteal nerve, while high-density EMG identified persistent deficits in spatial and temporal organization despite clinical improvement. Load-bearing and assisted-task studies showed that cane use and balance challenges modulate abductor demand yet continue to expose asymmetries and elevated stabilization requirements. Nonetheless, comparability across investigations remains limited because few studies adopted standardized normalization procedures or reproducible locomotor tasks. **Conclusions**: Neuromuscular recovery after THA appears incomplete and asymmetric, characterized by compensatory strategies not detectable through clinical or kinematic assessments alone. Improved diagnostic sensitivity and clinical applicability will require protocol standardization and the broader adoption of advanced EMG approaches.

## 1. Introduction

Hip osteoarthritis is a highly prevalent, progressively disabling musculoskeletal disorder whose burden increases sharply with age. The hip joint is a biomechanical hub for load transmission and efficient locomotion; degenerative changes at this level have disproportionate consequences for pain, mobility, and loss of independence [1]. Contemporary population-based estimates indicate that the condition affects tens of millions of individuals worldwide, with approximately 36 million prevalent cases and about 1.8 million new cases annually. Both incidence and prevalence have exhibited a sustained upward trajectory over recent decades [2,3].

Total hip arthroplasty (THA) is a widely adopted procedure in the context of hip osteoarthritis and one of the most commonly performed surgeries globally. It is estimated that between 6% and 16% of the general population will undergo THA at some point in their lifetime [3].

In routine clinical practice, postoperative monitoring after THA primarily relies on patient-reported outcome measures assessing pain, perceived function, and quality of life [4,5]. This is supplemented by a standard radiographic assessment of implant position and integrity [6]. Radiographic follow-up typically includes anteroposterior and lateral projections of the hip to evaluate component alignment, fixation, and stability, while computed tomography may be used selectively to more accurately assess prosthetic component orientation [7].

Overall, THA effectively reduces pain, improves functional outcomes, and enhances patient-reported quality of life, generally yielding satisfactory clinical and radiographic results [4,5]. However, favorable clinical scores and satisfactory imaging findings do not necessarily reflect full recovery of neuromuscular function [8]. These approaches are designed to capture symptomatic relief and structural implant integrity but provide limited insight into muscle activation patterns, intermuscular coordination, and motor control strategies during functional activities.

Importantly, many patients exhibit degenerative alterations in periarticular muscles and tendons prior to surgery because of prolonged disuse, compensatory movement strategies, and disease-related degeneration [9,10]. The surgical procedure itself may further induce neuromuscular modifications related to tissue trauma, periarticular muscle dissection, altered joint mechanics, and potential vascular compromise [11,12]. Additional risk factors include traction, irritation, or partial injury of periarticular nerves, with incidence varying according to the surgical approach employed [13,14,15,16]. As a result, neuromuscular activation patterns may remain altered after THA even when the procedure is technically successful and structural outcomes appear satisfactory [17].

From this perspective, THA should not be considered solely as a structural reconstruction, but rather as a major biomechanical and neuromuscular transition. In this context, electromyography (EMG) has become an essential tool for studying neuromuscular adaptations after THA. EMG records the electrical activity generated by skeletal muscles and their motor units during activation, allowing direct assessment of muscle activation and neuromuscular control.

Needle EMG enables detailed evaluation of motor unit integrity and identification of denervation or reinnervation phenomena [18]. Early investigations using this approach during the initial postoperative phase revealed impaired voluntary activation and reduced recruitment capacity of the hip abductors [19,20]. Subsequently, surface EMG has been applied to characterize muscle activation patterns during movement, providing quantitative information on timing, amplitude, and intermuscular coordination during functional tasks [21].

These analyses showed that abnormalities in muscle activity—often still present several months after surgery—involve not only the abductors but also other hip-related muscles during gait and load-bearing activities [22,23]. More recent developments, such as high-density surface electromyography (HD-sEMG) and other multimodal EMG-based analytical approaches, have demonstrated that postoperative neuromuscular reorganization extends beyond the hip region and involves coordinated adaptations across the entire limb and trunk [24]. Notably, these non-physiological patterns can persist well beyond pain resolution and basic functional recovery and may remain detectable up to one year or more after surgery [25]. The widening gap between biomechanical recovery and neuromuscular reorganization underscores a significant blind spot in current postoperative evaluation models.

Despite these insights, the nature, consistency, and clinical implications of reported EMG alterations remain difficult to interpret. A major source of uncertainty lies in the substantial methodological heterogeneity of available studies. Existing investigations range from treadmill and overground gait analysis to static load tests and diagnostic maneuvers such as the Trendelenburg test [22,26], and more recently have included EMG assessments during standardized rehabilitation or mobilization exercises performed in lying, sitting, or standing positions [24]. This diversity in motor tasks, experimental conditions, and measurement protocols makes it difficult to identify robust and reproducible neuromuscular patterns.

Such heterogeneity is further compounded by differences in electrode placement, filtering methods, and normalization strategies—including maximum voluntary isometric contraction (MVIC), percentage of maximum voluntary contraction (%MVC), and root mean square (RMS) values—as well as variability in the muscles examined, postoperative time points, surgical approaches, and prosthesis types. A further limitation is that many investigations have focused primarily on individual muscles, most commonly the gluteus medius (GMED), rather than examining the broader organization of motor control. As a result, intermuscular coordination, bilateral load distribution, and compensatory strategies influenced by contralateral pathology or previous arthroplasty remain only partially understood.

In this context, a systematic reappraisal of the EMG literature after THA is warranted. The main objectives of this review are to determine the extent to which postoperative EMG patterns resemble those of healthy individuals or preoperative conditions, to identify the most consistent neuromuscular alterations, and to explore how factors such as surgical approach, nerve integrity, and load management contribute to the persistence of dysfunction. Another aim is to translate this evidence into practical clinical recommendations and outline future research directions that may deepen understanding of motor recovery after THA.

## 2. Materials and Methods

### 2.1. Guidelines and Protocol

This systematic review was conducted in accordance with the Preferred Reporting Items for Systematic Reviews and Meta-Analyses (PRISMA) 2020 guidelines [27] (Table S1). The methodological protocol, including the search strategy, eligibility criteria, screening procedures, data extraction framework, and planned approach to synthesis, was defined as a priori and preregistered on the Open Science Framework (OSF; DOI: 10.17605/OSF.IO/DA5JT).

### 2.2. Search Strategy

A comprehensive literature search was conducted in PubMed/MEDLINE, EMBASE, Scopus, Web of Science Core Collection, CINAHL, IEEE Xplore, Cochrane CENTRAL, and Google Scholar (first 200 records). The search strategy combined controlled vocabulary and free-text terms related to THA, surface and needle EMG techniques, and both dynamic and static functional tasks. Search strings were adapted to the syntax and indexing structure of each database. No language restrictions were applied. The search was last updated in December 2025. To ensure transparency and reproducibility, the complete electronic search strategies for each database, including the exact search strings, Boolean operators, and applied filters, are reported in the Supplementary Materials (Table S2).

### 2.3. Eligibility Criteria

Eligible studies were original peer-reviewed research involving adult participants undergoing primary or revision THA and reporting quantitative EMG analyses of hip or lower-limb muscles. Assessments could include dynamic locomotor tasks such as gait or stair ambulation, static or isometric contractions, Trendelenburg or load-modified conditions, or intraoperative neuromonitoring. Studies were required to report at least one interpretable EMG parameter, such as activation timing, amplitude (normalized or raw), motor unit characteristics, frequency-domain features, or advanced EMG descriptors. Studies were excluded if they used EMG solely for biofeedback, provided no analyzable EMG data, involved mixed populations without separable THA results, or consisted of non-original material such as conference abstracts, theses, or narrative reviews. In addition, studies employing needle EMG exclusively for intraoperative neuromonitoring—not for quantitative assessment of postoperative muscle activation—were excluded.

### 2.4. Screening Process

All records retrieved from the search were imported into a reference manager, duplicates were removed, and the remaining records were screened independently by two reviewers based on the title and abstract. Full texts judged potentially eligible were then retrieved and assessed in detail according to the predefined criteria. Disagreements were resolved through discussion and consensus. The flow of studies through the screening stages is reported in Section 3 and illustrated in the PRISMA flow diagram.

### 2.5. Data Extraction

Data extraction was performed independently by two reviewers, and any discrepancies were resolved through consensus. For each included study, we recorded the study design and sample characteristics, as well as the postoperative follow-up intervals considered. We also extracted information on the surgical approach adopted—classified as posterolateral, direct lateral, direct anterior, minimally invasive anterior, or other variants—and on the specific muscles examined, with particular attention to the GMED, gluteus maximus (GMAX), tensor fasciae latae (TFL), rectus femoris (RF), biceps femoris (BF), semitendinosus (ST) and sartorius (S).

Details regarding the functional protocols were documented, including the type of task performed, walking speed, support conditions, and any load modifications. The EMG methodology was also collected, distinguishing between surface, needle, fine-wire, and multimodal neurophysiological monitoring techniques. Normalization procedures were recorded when available, such as the use of MVIC, %MVC, RMS normalization, or the absence of normalization.

Finally, for each study we extracted the analyzed EMG parameters—encompassing timing metrics, amplitude (normalized or raw), motor unit action potential (MUAP) characteristics, and frequency-domain features—and summarized the primary EMG-related results together with the authors’ main interpretations and conclusions.

### 2.6. Methodological Quality Assessment

The methodological quality was evaluated using relevant Joanna Briggs Institute (JBI) critical appraisal tools adapted to the specific features of EMG studies [28]. Attention was given to the transparency and reproducibility of EMG acquisition, preprocessing, and normalization procedures, as well as the robustness and appropriateness of timing detection and amplitude quantification methods. Other areas of focus included adherence to established standards for needle, surface, and quantitative EMG, and the clarity and completeness of statistical reporting.

Based on the overall appraisal, the studies were classified qualitatively into four categories of methodological quality (very high, high, good, or medium). Higher ratings reflected greater methodological rigor and completeness, whereas lower ratings indicated increasing limitations or incomplete reporting.

Quality assessments were performed independently by two reviewers, and discrepancies were resolved through consensus. These appraisals informed the interpretation of findings but did not determine study inclusion.

## 3. Results

### 3.1. Study Selection

**Figure 1:**
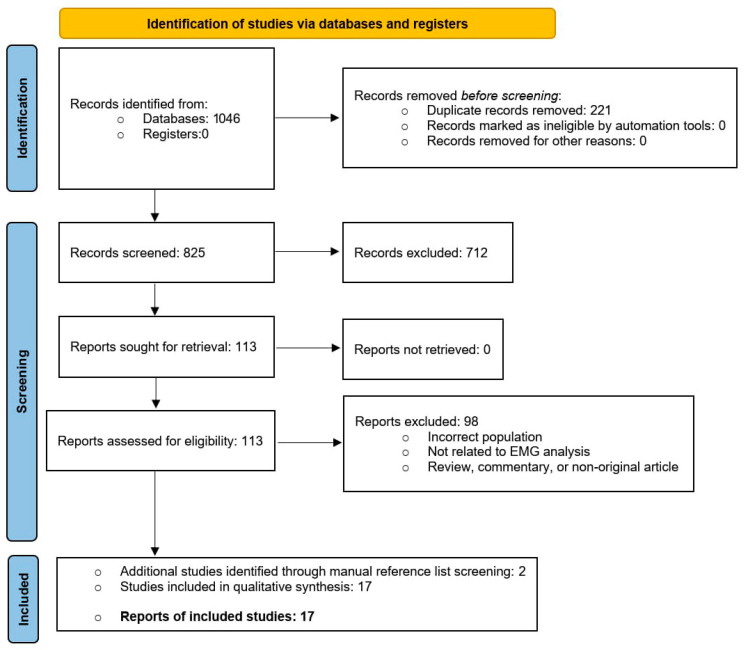
PRISMA Flow Diagram.

A total of 1046 records were identified through database searching. After removal of 221 duplicates, 825 unique records underwent title and abstract screening. Of these, 712 were excluded because they did not involve THA populations, did not include EMG outcomes, or adopted ineligible designs. The remaining 113 full-text articles were assessed for eligibility; none were excluded due to retrieval failure. Following full-text evaluation, 98 studies were excluded because they investigated non-THA samples, lacked analyzable EMG parameters, provided in-sufficient methodological detail, or were classified as non-original re-search (review, commentary, or technical note). A total of 15 studies met all inclusion criteria, and two additional eligible study were identified through manual reference screening, resulting in 17 studies included in the qualitative synthesis. The full selection process is presented in Figure 1 (PRISMA Flow Diagram).

### 3.2. Methodological Quality Assessment

The methodological quality varied across the included studies, as summarized in Table 1. Investigations focusing on activation timing, such as those by Agostini [29], Vogt [30], Horstmann [22], and Ajemian [31], generally provided clear descriptions of onset/offset detection and burst quantification, although not all incorporated amplitude normalization, limiting cross-study comparability. Studies employing MVIC-normalized amplitude, including Martinez [32], Bernard [23], Kopeć [26], and Robbins [33], demonstrated stronger methodological consistency, particularly in relation to electrode placement, preprocessing pipelines, and normalization strategies. Robbins’ integration of waveform principal component analysis represented one of the most analytically sophisticated approaches [33].

Needle EMG and quantitative EMG investigations—particularly those by Baker & Bitounis [20], Moreschini [19], Chomiak [16], and Khalifa [34]—showed high technical rigor, supported by detailed reporting of motor-unit morphology, spontaneous activity, and recruitment characteristics. Advanced EMG descriptors used by Ippolito [30] (full width at half maximum (FWHM)) and Robbins [33] (principal component analysis (PCA)) added valuable analytical depth, though these methods were less widely adopted. HD-sEMG, as applied in Morsch et al. [24], further expanded methodological sophistication by incorporating spatial activation mapping, spectral features, and coordination indices within a longitudinal framework.

Conversely, studies relying on raw, non-normalized amplitude or qualitative pattern description—such as Novo [25]—showed good technical execution but lower evidence strength due to limited reproducibility and reduced comparability. The main sources of heterogeneity across studies were differences in normalization strategy, preprocessing detail, sampling consistency, and the complexity of the analytical frameworks.

Despite this variability, all included studies met the minimum standards required for EMG acquisition and reporting, allowing for their retention in the review and enabling structured synthesis across methodological subgroups.

**Table 1:** Summary of methodological characteristics of the included studies, detailing the primary EMG domain, specific metrics assessed, and overall methodological quality rating.

Study	Primary EMG Domain	Actual EMG Metrics Assessed	Methodological Quality
[29]	Timing	Onset/offset, burst duration, number of activations using statistical detectors across consecutive gait cycles	High
[30]	Timing	GMED onset/cessation normalized to stride time (% stride); pre-activation duration	Good
[22]	Timing + amplitude	Band-pass filtered, rectified EMG; RMS envelope; amplitude normalized to standardized submaximal contractions; time-normalized intensity curves	Good
[25]	Raw amplitude + qualitative timing	Qualitative EMG pattern analysis across normalized gait cycle; extra activations; phase shifts; raw (non-normalized) amplitude	High technical quality/low evidence level
[32]	MVIC-normalized amplitude + timing	Surface EMG normalized to MVIC for GMED, GMAX, TFL, sartorius; comparison of normalized activation patterns during gait	High
[35]	Advanced timing (FWHM/CoA)	FWHM and Center of Activation (CoA) in polar coordinates across the gait cycle	High
[20]	Needle EMG (denervation)	Fibrillation potentials, positive sharp waves, qualitative grading of denervation at 2 w and 3 m; TFL targeted sampling	High technical quality
[19]	Needle EMG (MUAP recruitment)	Qualitative MUAP recruitment pattern, fibrillations/PSW, assessment of “poor” vs. “valid” tracings; comparison across approaches	Medium–high
[33]	MVIC-normalized amplitude + PCA	Band-pass 20–500 Hz; rectified; low-pass 6 Hz envelope; normalized to MVIC; waveform PCA for GMED, GMAX, quadriceps, hamstrings, TFL	Very high
[36]	Surface + fine-wire EMG + spectral analysis	Burst timing, MdPF, relative amplitude patterns for GMED/TFL/iliopsoas; fine-wire morphology	High technical quality
[34]	Quantitative EMG (QEMG)	MUAP amplitude, duration, number of phases; spontaneous activity; interference pattern; serial QEMG changes	Very high
[31]	Timing	Burst duration during stance; high-pass 25 Hz; EMG “on” >15% peak; 10% gait-cycle bins; comparisons with/without cane	Medium
[23]	MVIC-normalized amplitude (static stance)	EMG from GMAX, GMED, TFL, sartorius; 20–450 Hz filtering; rectified/smoothed; normalized to MVIC; comparison across stance tasks	Medium–high
[26]	MVIC-normalized amplitude (Trendelenburg)	GMED %MVIC during full- vs. low-load Trendelenburg; pre- and post-THA comparisons	Medium–high
[37]	RMS amplitude (functional EMG during gait)	RMS linear envelope (55 ms); mid-stance amplitude; normalized to MVC; comparison of cane-use and load-carriage	Medium
[16]	Needle EMG (approach-specific denervation)	Rates of denervation in GMED, GMAX, TFL across anterolateral, transgluteal, and posterior approaches; MUAP abnormalities	High technical quality
[24]	High-density EMG (multidimensional functional indices)	64-channel HD-sEMG; spatial activation maps; spectral features; coordination indices; activation consistency; longitudinal change	Very high (advanced methodology + longitudinal design)

Abbreviations: EMG—Electromyography; GMED—Gluteus Medius; GMAX—Gluteus Maximus; TFL—Tensor Fasciae Latae; MVIC/MVC—Maximal Voluntary (Isometric) Contraction; MUAP—Motor Unit Action Potential; QEMG—Quantitative Electromyography; RMS—Root Mean Square; FWHM—Full Width at Half Maximum; CoA—Center of Activation; PCA—Principal Component Analysis; MdPF—Median Power Frequency; PSW—Positive Sharp Waves; HD-sEMG—High-Density Surface Electromyography.

### 3.3. Alterations in EMG Patterns During Gait After THA

Six studies [22,25,29,30,32,35] examined EMG activity during walking after THA. Follow-up intervals ranged from approximately six weeks to 12 months, with one long-term case assessed more than 10 years post-surgery [25]. Despite differences in locomotor settings (overground vs. treadmill, self-selected vs. controlled speed) and normalization methods (MVIC, standardized isometric contractions, or no normalization), all studies analyzed either timing parameters (onset, offset, burst duration) or normalized amplitude, and all reported deviations from physiological patterns.

GMED emerged as the most consistently altered muscle. According to studies, it showed delayed onset [30], reduced pre-activation before heel strike [29], and prolonged activation during stance [25,29]. These abnormalities were observed in both cross-sectional comparisons with healthy controls [30,32,35] and longitudinal pre–post analyses [22], indicating that the abductor complex fails to fully recover its anticipatory and stabilizing role during gait.

Posterior biarticular muscles, particularly BF and ST, displayed markedly sustained activity during late stance in both longitudinal [22] and cross-sectional [25] studies. This pattern suggests a compensatory use of the hamstrings as secondary stabilizers, potentially offsetting persistent abductor weakness or inefficient hip extension. Abnormalities were also reported in the flexors and lateral stabilizers, including RF, TFL, and sartorius, with patterns that varied according to surgical approach. In studies focusing on lateral or posterolateral approaches [22,29,30], these muscles tended to exhibit prolonged bursts with delayed peaks, whereas in anterior approaches [32,35] the abnormalities were characterized more by reduced amplitude and less well-defined temporal profiles. Despite these differences, both patterns represent deviations from normal intermuscular coordination.

Overall, the available gait studies show that improvements in walking capacity and spatiotemporal parameters do not coincide with normalization of EMG patterns. Persistent delays in GMED activation, hamstring overactivity, and flexor inefficiencies suggest a non-physiological recovery trajectory extending beyond the first postoperative year. Full details are presented in Table 2.

### 3.4. Surgical Approach and EMG Patterns

Four studies directly compared EMG outcomes across surgical approaches [16,19,20,33]. Two early investigations used needle EMG to assess postoperative denervation and voluntary recruitment [19,20], while Chomiak et al. [16] expanded this approach to three surgical techniques, and Robbins et al. [33] examined MVIC-normalized surface EMG during gait one year after THA.

In the early postoperative period, both Moreschini and Baker reported a higher incidence of denervation potentials in TFL and impaired voluntary recruitment of GMED after lateral-based approaches compared with posterolateral access [19,20]. These changes implicated branches of the superior gluteal nerve, which are more exposed during lateral dissections. Neither study observed spontaneous activity in GMAX or sartorius, suggesting that denervation was localized to specific gluteal-muscle nerve branches.

Chomiak et al. [16] provided one of the most comprehensive comparative analyses of gluteal nerve involvement, examining 70 patients before and 3–9 months after surgery with anterolateral, transgluteal, or posterior approaches. Needle EMG revealed clear approach-specific patterns: the anterolateral approach resulted in the highest rate of TFL denervation (73%) but largely preserved GMED and GMAX; the transgluteal approach produced the most frequent partial denervation of GMED (81.8%) and also affected TFL and GMAX; the posterior approach showed higher rates of GMAX involvement (71.4%) and moderate GMED changes (53.3%). Notably, despite substantial EMG abnormalities, clinical abductor strength was not significantly reduced, supporting the presence of compensatory strategies that mask underlying deficits.

At a later follow-up, Robbins et al. [33] found no significant differences in mean activation amplitude between lateral and posterior approaches during gait. However, the underlying neuromuscular strategies diverged: lateral approaches showed increased GMED activity during stance, whereas posterior approaches were characterized by greater GMAX and hamstring activation. Both patterns differed from those observed in healthy controls, reflecting persistent deviations in neuromuscular coordination rather than complete recovery.

Taken together, these four studies consistently indicate that lateral-based approaches carry greater early risk to branches of the superior gluteal nerve, particularly those supplying TFL and GMED, while posterior approaches tend to spare TFL but may impact GMAX. Over time, however, approach-specific patterns tend to be replaced by more global compensatory strategies, and clinical performance may not reliably mirror the extent of EMG-detected abnormalities. Comparative results are summarized in Table 3.

### 3.5. EMG Approaches: Needle and High-Density EMG

Three studies employed advanced EMG techniques—needle EMG, fine-wire EMG, or HD-sEMG—to provide a deeper characterization of neuromuscular status after THA [24,34,36].

Khalifa et al. [34] documented clear early postoperative abnormalities six weeks after a modified direct lateral approach, including fibrillation potentials, positive sharp waves, and prolonged MUAP duration in GMED, GMIN, and TFL, with partial resolution by twelve weeks. These findings were consistent with transient involvement of branches of the superior gluteal nerve, indicating that neurophysiological disturbances may persist even when clinical presentation appears satisfactory.

Chopra et al. [36] combined surface and fine-wire EMG with frequency-domain analysis to assess neuromuscular organization up to one year after THA performed through a direct anterior approach. Despite clinical improvement, none of the evaluated muscles—including GMED, GMAX, iliopsoas, and paraspinals—showed fully normalized temporal or spectral profiles, suggesting ongoing reorganization of motor control.

A third study applied HD-sEMG to provide a high-resolution assessment of neuromuscular recovery. Morsch et al. [24] used a wearable 64-channel array to longitudinally track GMED activity, deriving multidimensional indices of activation, spatial efficiency, coordination, and pattern fidelity. These indices frequently revealed persistent compensatory activation or incomplete normalization of spatiotemporal patterns, even in participants reporting substantial clinical improvement.

Taking together, evidence from needle EMG, fine-wire EMG, and HD-sEMG indicates that neuromuscular recovery after THA is often incomplete, asymmetric, and characterized by persistent compensatory strategies, even when clinical and radiographic outcomes appear satisfactory. Detailed results are presented in Table 4.

### 3.6. EMG Activity Under Load-Bearing and Assisted Conditions

Four studies investigated EMG activity of hip abductors and stabilizers under conditions that alter load distribution or stabilizing demands, including walking with a cane, the Trendelenburg test, and static balance tasks [23,26,31,37].

Neumann et al. [32] found that using a contralateral cane significantly reduced GMED activity on the operated side during mid-stance, consistent with the mechanical unloading of the hip abductor moment. In contrast, combining an ipsilateral cane with a contralateral weight led to a marked increase in GMED activation, indicating that subtle changes in external support and load placement can substantially modify the neuromuscular demands imposed on the operated hip.

Ajemian et al. [31] reported that contralateral cane use shortened the burst duration of GMED and TFL, whereas walking without assistive devices preserved a temporal pattern similar to the preoperative one, even eight months after surgery. These findings suggest that external assistance may modulate neuromuscular activation without necessarily promoting normalization of the underlying control strategies.

During the Trendelenburg test, Kopeć et al. [26] observed a reversal of typical abductor activation patterns at six months: GMED activity decreased on the operated limb under full load, while it increased on the contralateral limb, especially in patients with bilateral osteoarthritis. This redistribution suggests an unstable strategy in which the contralateral limb assumes a disproportionate share of the stabilizing demand.

Bernard et al. [23] studied bipedal and unipedal stance 6–8 weeks after anterior THA and found high levels of activation in GMED, GMAX, and TFL, indicating that postural control relies on elevated muscular effort even in relatively simple static conditions. These data support the hypothesis that compensatory patterns are not limited to dynamic tasks but also extend to everyday balance and load-bearing situations.

Taken together, these studies show that EMG organization after THA is highly sensitive to how load is managed and how external support is used. The compensation strategies that emerge appear to reflect enduring neuromuscular adaptations rather than transient transitional states. Complete results are summarized in Table 5.

## 4. Discussion

The methodological appraisal demonstrated that all included studies met acceptable standards for EMG acquisition and reporting, despite notable heterogeneity in the normalization procedures, preprocessing, and analytical depth. Within this context, the findings of the review indicate that neuromuscular recovery after THA is slower and more complex than suggested by clinical scales, radiographic findings, or basic gait parameters. Across 17 studies using surface, needle, fine-wire, or HD-sEMG, consistent abnormalities were documented in activation timing, amplitude regulation, and motor unit behavior for months—and in some cases up to a year or more—after surgery. These results collectively suggest that the reorganization of hip muscle activation is not a simple byproduct of restored joint mechanics, but rather reflects a prolonged process of neural adaptation, altered load management, and persistent compensatory strategies.

### 4.1. EMG Alterations During Gait: A Non-Physiological Recovery Trajectory

The available gait studies reveal a clear dissociation between clinical recovery and neuromuscular normalization. Walking ability, pain, and basic functional measures often improve within the first months after THA [22,32], yet EMG patterns remain markedly abnormal at all examined follow-up points. A similar dissociation has been documented in total knee arthroplasty, where improvements in pain, range of motion, and spatiotemporal parameters coexist with persistent abnormalities in timing and co-contraction of key muscle groups [38]. This convergence across joints suggests that standard clinical indices substantially underestimate the persistence of neuromuscular dysregulation after joint replacement.

GMED appears to be a central element in this process. All studies assessing its activation timing [29,30,35] reported delayed onset and reduced pre-activation, indicating a long-standing deficit in anticipatory control of pelvic stability. Even at very long-term follow-up [25], GMED bursts remain prolonged in phases where activity would normally decline, pointing to a reorganized motor control strategy rather than a transient postoperative impairment.

Alterations in posterior muscles—particularly the biceps femoris and semitendinosus—further reinforce the presence of compensatory recruitment. Posterolateral and lateral approaches consistently show prolonged or persistent hamstring activation during late stance [22,29], while long-term observations also document continuous semitendinosus activity across the gait cycle [25]. This sustained activation pattern suggests that the hamstrings may adopt an atypical stabilizing role, compensating either for residual abductor weakness or for reduced hip extensor efficiency, the latter supported by decreased sagittal-plane hip moments in anterior-approach cohorts [32]. Such compensations likely alter load distribution across both the hip and lumbar spine.

Different, but comparably dysfunctional, activation patterns are also observed in the flexor and lateral muscle groups, namely the RF, S, and TFL. In lateral and posterolateral approaches [22,29], these muscles frequently exhibit prolonged bursts and delayed peaks, reflecting an expanded contribution to frontal-plane stabilization. In contrast, anterior approaches [32,35] are characterized by reduced activation amplitudes and poorly defined temporal profiles, suggesting diminished neuromuscular drive and blunted phase specificity. Although the specific manifestations differ, both configurations indicate incomplete restoration of intermuscular coordination and highlight the persistent influence of approach-specific biomechanical perturbations.

Overall, the gait literature strongly suggests that restoration of pain-free walking and acceptable spatiotemporal parameters does not equate to normal neuromuscular control. Persistent delays, compensatory hamstring activity, and approach-specific dysfunctions in flexor–lateral muscles support the need for rehabilitation strategies that explicitly target timing, coordination, and pelvic stability rather than focusing solely on gross functional outcomes.

### 4.2. Influence of Surgical Approach: Early Divergence, Late Convergence

Studies comparing surgical approaches reveal a two-phase pattern: marked early differences followed by partial convergence over time. In the initial postoperative period, Moreschini and Baker consistently showed that lateral-based approaches produce greater impairment of the abductor complex than posterolateral access, with higher rates of TFL denervation and reduced voluntary recruitment of GMED—findings that point to the vulnerability of superior gluteal nerve branches during lateral dissections [19,20]. Chomiak et al. [16] reinforced this perspective by demonstrating approach-specific denervation profiles: anterolateral access predominantly affecting TFL, transgluteal approaches more frequently compromising GMED, and posterior access showing greater involvement of GMAX. Despite these differences, clinical abductor strength was often preserved, suggesting early compensatory activation.

At a longer follow-up, the picture becomes more nuanced. Robbins et al. [33] observed no major differences in overall activation amplitude between lateral and posterior approaches at 12 months, although the underlying strategies diverged: lateral approaches showed increased GMED activation during stance, whereas posterior approaches relied more on GMAX and hamstrings. These patterns remain distinct from each other and from healthy controls, indicating that long-term neuromuscular organization reflects adaptive compensation rather than a persistent imprint of the surgical route.

In summary, lateral-based approaches impose a higher early neuromuscular burden, with more pronounced deficits in abductor-related muscles, whereas long-term outcomes across approaches shift toward different—but equally non-physiological—compensatory patterns. This highlights the need for targeted early rehabilitation after lateral access and for later interventions focused on correcting the specific compensatory strategies that emerge over time.

### 4.3. Evidence from Needle and High-Density EMG

Needle EMG provides direct insight into the neurophysiological consequences of THA by revealing denervation signs, altered motor unit recruitment, and early reinnervation processes that remain largely undetectable with clinical examination or conventional surface EMG, which primarily reflects global activation timing and amplitude rather than motor unit–level neural integrity [39,40]. Khalifa et al. [34] demonstrated that postoperative abnormalities—including fibrillation potentials, positive sharp waves, and prolonged MUAP duration—are common six weeks after a lateral-based approach, with partial resolution by twelve weeks. These findings suggest that transient involvement of superior gluteal nerve branches may occur more frequently than clinically recognized, often following a reversible neurapraxic pattern that remains clinically silent.

Complementary evidence is provided by studies using fine-wire EMG combined with frequency-domain analysis, which occupies an intermediate position between needle and surface EMG. While still invasive, fine-wire EMG enables selective recording from deep or overlapping muscles during controlled functional tasks. Chopra et al. [36] showed that even one year after direct anterior THA, temporal organization and spectral characteristics of muscle activation remain atypical across several hip and trunk muscles. These persistent deviations indicate that motor control reorganization extends well beyond the timeframe in which patient-reported outcomes, strength measures, and basic gait parameters typically appear normalized, helping to explain discrepancies frequently observed between clinical recovery and residual movement inefficiency.

HD-sEMG adds a complementary, non-invasive, high-resolution perspective by shifting the analytical focus from single motor units to the spatial organization and coordination of muscle activation. Unlike conventional surface EMG, HD-sEMG enables the assessment of activation heterogeneity, spatial efficiency, and pattern fidelity across the muscle surface during dynamic tasks. Longitudinal data from Morsch et al. [24] revealed that indices reflecting spatial coordination and activation efficiency often remain abnormal despite improvements in patient-reported outcomes. This dissociation underscores that neuromuscular recovery after THA does not necessarily parallel subjective improvement or gross functional restoration.

Taken together, evidence from needle, fine-wire, and high-density EMG supports a multifaceted model of neuromuscular adaptation after THA, characterized by early neural vulnerability, incomplete normalization of activation timing and spectral features, and persistent alterations in spatial coordination. While studies using conventional surface EMG consistently report delayed or reduced muscle activation, advanced EMG techniques reveal that the quality of neuromuscular control—how muscles are recruited, coordinated, and integrated into motor strategies—may remain compromised even when activation is present. From a clinical and research perspective, these findings suggest that neuromuscular recovery after THA should not be interpreted solely in terms of muscle “on–off” behavior, but rather as a gradual and often incomplete restoration of efficient neuromotor organization.

### 4.4. EMG Under Load-Bearing Conditions: Compensation Beyond Gait

Weight-bearing tests and tasks that manipulate external support shed light on how the neuromuscular system manages load after THA. In this domain, the use of canes and the distribution of weight between limbs emerge as particularly important determinants of EMG organization.

The reduction in GMED activity with contralateral cane use observed by Neumann and Ajemian is consistent with biomechanical models showing that contralateral support reduces the abductor moment required at the operated hip [32,37]. Conversely, the combination of an ipsilateral cane and contralateral load markedly increases GMED activation, highlighting how small changes in external support can dramatically modulate muscular demand.

The Trendelenburg findings reported by Kopeć et al. [26] further demonstrate that months after surgery, abductor activation is often redistributed toward the contralateral limb, particularly when that limb is itself affected by osteoarthritis. Rather than restoring symmetrical load sharing, the system appears to favor strategies that shift the stabilization burden to the less symptomatic or more mechanically favorable side.

Bernard et al. [23] added another layer by demonstrating that even in static conditions—bipedal and unipedal stance—GMED, GMAX, and TFL activation remains high shortly after surgery, suggesting that postural stability is achieved at the cost of increased muscular effort. These findings support the view that compensatory patterns after THA are not limited to dynamic locomotion but extend across the spectrum of everyday weight-bearing tasks.

Collectively, the evidence indicates that load modulation, external assistance, and postural control strategies play a central role in shaping postoperative EMG patterns. If not specifically addressed in rehabilitation, these compensations may become entrenched, potentially contributing to residual fatigue, altered loading of adjacent joints, and suboptimal functional outcomes.

### 4.5. Methodological Limitations

Several limitations should be acknowledged when interpreting the findings of this systematic review. First, electromyographic outcomes are intrinsically task-specific and strongly influenced by locomotor context, including walking modality (e.g., treadmill vs. overground), speed, load distribution, and the use of external support. Accordingly, EMG findings must be interpreted within the specific task in which they were obtained, and heterogeneity in locomotor contexts intrinsically limits direct cross-study comparability. This issue reflects the fundamental property of EMG rather than a shortcoming of individual investigations.

Second, the number of available studies investigating neuromuscular function after total hip arthroplasty using EMG remains limited, and no two studies adopted identical experimental tasks, protocols, or follow-up schedules. Therefore, the present synthesis necessarily relies on qualitative interpretation of individual studies rather than on quantitative aggregation or task-level meta-analysis. This limitation reflects the current state of the literature and underscores the need for more standardized experimental paradigms.

Third, substantial methodological heterogeneity was observed across studies with respect to EMG acquisition parameters, signal processing techniques, normalization procedures, muscle selection, and outcome definitions. Differences in electrode placement, filtering strategies, and analytical domains further constrain direct comparisons and preclude formal quantitative synthesis.

Fourth, some interpretative considerations necessarily draw on individual studies, including isolated reports with very long follow-up intervals exceeding ten years post-surgery. These findings should therefore be regarded as exploratory and hypothesis-generating rather than confirmatory, and they were not used to support the core conclusions of the review.

Nevertheless, despite the limited number of studies, heterogeneous task designs, and reliance on qualitative synthesis, a consistent pattern emerges across the literature. Neuromuscular recovery after total hip arthroplasty frequently appears incomplete and asymmetric and is characterized by persistent compensatory activation strategies that are not detectable through clinical or kinematic assessments alone. The recurrence of this qualitative finding across diverse experimental paradigms and timepoints supports the robustness of the overarching interpretative message of the review.

### 4.6. Practical Implications

The EMG evidence summarized in this review has several practical implications for postoperative assessment and rehabilitation.

First, the dissociation between clinical recovery and neuromuscular normalization suggests that routine follow-up should not rely exclusively on pain scores, ROM, and general functional tests. Targeted evaluation of muscle activation timing, amplitude, and symmetry—at least in high-risk or symptomatic patients—may help identify persistent deficits that are invisible to standard clinical examination.

Second, the central role of GMED and the consistent presence of hamstring compensations argue in favor of rehabilitation programmers that prioritize abductor pre-activation, pelvic stability, and selective recruitment of hip extensors and stabilizers. Exercises that challenge frontal-plane control and anti-rotation in both dynamic and static conditions may be particularly beneficial.

Third, the marked sensitivity of EMG patterns to load distribution and cane use implies that assistive devices should be prescribed and monitored as active elements of rehabilitation rather than as passive supports. Training patients to use contralateral support correctly and to gradually rebalance load between limbs may reduce the persistence of asymmetric compensations.

Finally, in patients with bilateral osteoarthritis or previous contralateral arthroplasty, special attention should be paid to the “apparently healthy” limb, which may become the primary source of asymmetry and overuse. EMG-informed protocols could help tailor rehabilitation to these complex scenarios.

### 4.7. Future Directions

The available literature highlights substantial methodological heterogeneity and the need for more sensitive and standardized approaches to characterize neuromuscular reorganization after THA. Several directions for future research can be identified.

First, there is a clear need to standardize EMG protocols, including a minimal set of key muscles (e.g., GMED, GMAX, TFL, RF, BF, ST), shared definitions for onset and offset detection, robust criteria for burst duration, and agreed normalization procedures (e.g., MVIC or standardized isometric tasks). Reproducible locomotor tasks and clearly defined static or load-modified paradigms should be adopted to enable meaningful comparisons across studies.

Second, higher-resolution methods such as HD-EMG, spatiotemporal activation maps, and spinal maps of motor output should be more widely used to infer segmental spinal contributions and to capture subtle reorganizations of motor commands that are not evident in traditional single-channel recordings [41,42,43,44]. These techniques can reveal microstructural changes in motor unit behavior and intermuscular coordination.

Third, the analysis of multi-muscle coordination through muscle synergy models represents a promising avenue. Approaches such as non-negative matrix factorization, space-by-time decompositions, and newer non-matrix frameworks [45,46] can provide insight into the structure, variability, and fragmentation of motor modules, helping to distinguish between adaptive and maladaptive reorganization.

Fourth, electrophysiological manifestation of muscular fatigue deserves greater attention. Fourier analysis, short-time Fourier transform (STFT), and wavelet-based methods are well suited for non-stationary EMG and can be applied to isometric and dynamic tasks [47,48,49]. Frequency-based indices such as median and mean power frequency, spectral compression, and related measures may offer complementary information on load-sustaining capacity and endurance after THA.

Finally, extended longitudinal studies with bilateral assessments are needed to determine whether EMG patterns ultimately normalize or whether stable compensatory strategies become the “new normal.” Such designs would help clarify the time course and determinants of neuromuscular reorganization and could inform the optimal timing, intensity, and content of rehabilitation interventions.

## 5. Conclusions

This systematic review demonstrates that neuromuscular recovery after THA proceeds at a markedly slower pace—and with greater complexity—than clinical and radiographic outcomes alone would suggest. Across surface, needle, fine-wire, and high-density EMG methodologies, persistent abnormalities in muscle activation were consistently documented well into the first postoperative year and, in some cases, beyond.

GMED emerged as the most robust indicator of impaired recovery, showing systematic delays in onset, reduced pre-activation, and prolonged activation during stance across dynamic tasks. Posterior musculature, particularly the hamstrings, frequently assumed an atypical stabilizing role, while anterior and lateral muscles displayed either inefficient or blunted activation patterns depending on the surgical approach. These disruptions were evident during gait, balance tasks, and load-bearing conditions, and they often persisted despite substantial improvements in pain and function.

Although lateral-based approaches were associated with greater early vulnerability—manifested by higher rates of transient denervation and disturbed recruitment of GMED and TFL—the long-term neuromuscular profiles of different approaches converged toward non-physiological compensatory strategies rather than complete normalization. Needle EMG and high-density EMG further revealed that electrophysiological abnormalities may persist even when structural and clinical evaluations appear satisfactory, underscoring a fundamental dissociation between joint reconstruction and neuromuscular integrity.

Taken together, the evidence indicates that neuromuscular recovery after THA is neither automatic nor guaranteed. Instead, it reflects a slow and incomplete process shaped by nerve integrity, compensatory motor behavior, and complex load-management adaptations. The absence of consistent normalization across EMG domains highlights the need for rehabilitation paradigms that explicitly target temporal coordination, abductor pre-activation, selective muscle recruitment, and symmetrical loading, rather than focusing exclusively on strength or ROM.

Within this framework, EMG emerges as an indispensable modality for detecting subtle yet clinically meaningful deficits, guiding postoperative rehabilitation, and refining our understanding of functional recovery following THA.

## Figures and Tables

**Table 2:** Surface EMG during gait after THA.

Study	Participants	EMG Aim	Follow-Up	Surgical Approach	Muscles	Task	EMG Outcomes	Key EMG Findings
[29]	20 THA, 20 controls	Characterize EMG gait alterations over 12 months	3, 6, 12 m	Posterolateral	TA, GL, RF, BF, GMED	10 m walkway + 150 s acquisition	Onset/offset, burst duration, activation count	TA onset ↑; GL offset ↑; BF & GMED burst duration ↑; RF unchanged
[22]	52 THA + 24 controls	Compare pre- vs. 6-month amplitude/timing	Pre-op, 6 m	Lateral transgluteal	RF, S, AM, TFL, BF, ST, GMAX, GMED	Treadmill 3 km/h	Amplitude normalized to submaximal contraction; burst timing	Pre-op EMG ↑ in all muscles; at 6 m RF/S/TFL/GMAX/GMED ↓; BF/ST ↑; incomplete normalization
[32]	15 THA (MIAA) + 12 controls	Amplitude and timing >1 year	12–18 m	MIAA	GMED, GMAX, TFL, S	14 m walkway	MVIC-normalized amplitude; timing	GMED/GMAX strength ↓; EMG patterns non-normalized
[25]	1 bilateral THA	Describe long-term EMG patterns	12 years	NR	TA, GL, RF, ST	6 m walk, 6MWT, treadmill	Raw amplitude; qualitative timing	Continuous TA activity; GL quasi-biphasic; ST persistent abnormal activation
[30]	14 THA + 10 controls	Quantify GMED timing at 6 weeks	31–46 days	Posterolateral	GMED bilat.	Treadmill	Cycle-normalized onset/offset	GMED onset delayed; reduced stance duration; ↑ pelvic tilt
[35]	14 THA (DAA) + 14 controls	Temporal distribution across three gait modes	Pre-op, 3 and 6 m	DAA	RF, TFL, GLME, GLMA	Forward, lateral, backward gait	FWHM & temporal distribution	GLME abnormalities persist to 6 m; partial normalization in lateral gait

Abbreviations: TA = tibialis anterior; GL = gastrocnemius lateralis; RF = rectus femoris; BF = biceps femoris; ST = semitendinosus; S = sartorius; AM = adductor magnus; TFL = tensor fasciae latae; GMED = gluteus medius; GLME/GLMA = medial/anterior GMED subdivisions; GMAX = gluteus maximus; MVIC = Maximum Voluntary Isometric Contraction; FWHM = Full Width at Half Maximum; MIAA = Minimally Invasive Anterior Approach; DAA = Minimally Invasive Direct Anterior Approach; ↑ = increase; ↓ = decrease.

**Table 3:** Surgical approach comparisons: EMG findings.

Study	Participants	Aim	Follow-Up	Approaches	Muscles	Task	EMG Outcomes	Key Findings
[20]	69 pts, 79 hips	Incidence of TFL denervation	2–3 w, 3 m	MDL vs. DL vs. PL	TFL (needle)	Needle EMG	Fibrillations/PSW	DL highest denervation; MDL moderate; PL low; almost resolved by 3 m
[19]	20 THA	MUAP recruitment differences	15 m	Hardinge vs. PL	GMED, GMAX, TFL	Needle EMG	MUAP quality	Hardinge: 5/10 poor recruitment (mainly GMED); PL: 1/10; no fibrillation
[33]	21 lateral, 21 posterior, 21 controls	Compare normalized gait EMG	~13 m	Lateral vs. Posterior	GMED, GMAX, TFL, RF, VM, VL, ST, BF	Over-ground gait	MVIC-normalized amplitude; PCA	Lateral: ↑ GMED; posterior: ↑ GMAX + hamstrings. Both ≠ controls
[16]	70 pts	Compare denervation across three surgical approaches	3–9 m	Anterolateral vs. Transgluteal vs. Posterior	GMED, GMAX, TFL	Needle EMG	Denervation incidence; MUAP abnormalities	Anterolateral: high TFL denervation (73%); transgluteal: highest GMED involvement (81.8%); posterior: greater GMAX involvement (71.4%); clinical abductor strength preserved despite EMG abnormalities

Abbreviations: TFL = tensor fasciae latae; GMED = gluteus medius; GMAX = gluteus maximus; RF = rectus femoris; VM = vastus medialis; VL = vastus lateralis; BF = biceps femoris; ST = semitendinosus; MVIC = Maximum Voluntary Isometric Contraction; PCA = Principal Component Analysis; PSW = Positive Sharp Wave; MUAP = Motor Unit Action Potential; MDL = Modified Direct Lateral; DL = Direct Lateral; PL = Posterolateral; AL = Anterolateral; TG = Transgluteal; ↑ = increase.

**Table 4:** Advanced EMG approaches after THA.

Study	Participants	Approach	Aim	Follow-Up	Muscles	Test	Outcomes	Key Findings
[34]	40 THA	Modified DL	Early postoperative QEMG	Pre-op, 6 w, 12 w	GMED, GMIN, TFL	Needle QEMG	MUAP parameters; PSW	6 w: 37.5% PSW; GMED ↓ amp & ↑ duration; recovery in 82% at 12 w
[36]	1 THA + 10 ctrls	DAA	Timing & MdPF during gait/stairs	Pre-op, 3 m, 12 m	GMED, GMAX, IP, TFL, RF, HS, LPS	Fine-wire + sEMG	Timing; MdPF	No full normalization at 12 m; persistent abnormalities
[24]	30 THA (subset of 63 TJA pts)	Lateral approach (Hip cases)	Longitudinal HD-sEMG monitoring; functional indices	Pre-op, 2 d, 4–5 d, 6 w, 3 m, 6 m	GMED (THA segment)	64-channel HD-sEMG	Spatial, temporal & spectral features; multidimensional functional indices	Persistent deficits in coordination & pattern fidelity; compensatory activation despite PROM improvement; HD-sEMG sensitive to subtle neuromotor abnormalities

Abbreviations: GMED = gluteus medius; GMIN = gluteus minimus; GMAX = gluteus maximus; TFL = tensor fasciae latae; IP = iliopsoas; RF = rectus femoris; HS = hamstrings; LPS = lumbar paraspinals; VM = vastus medialis; MUAP = Motor Unit Action Potential; PSW = Positive Sharp Wave; MdPF = Median Power Frequency; QEMG = Quantitative EMG; HD-sEMG = High-Density surface electromyography; DAA = Direct Anterior Approach; ↑ = increase; ↓ = decrease.

**Table 5:** EMG during load-bearing and support-assisted tasks.

Study	Participants	Aim	Follow-Up	Approach	Muscles	Task	EMG Outcomes	Key Findings
[37]	24 THA	Effect of cane & unilateral load	5–96 m	NR	GMED	Walking under 6 conditions	RMS normalized	Contralateral cane ↓ GMED 30–40%; ipsilateral cane ↑ GMED up to 80%
[31]	11 THA	Effect of cane on burst duration	Pre-op, 4 m, 8 m	NR	GMED, TFL, LH, RF, ES	Walking with/without cane	Burst duration (% stance)	Without cane: no normalization by 8 m; cane ↓ GMED/TFL duration
[26]	64 THA	Trendelenburg activation	Pre-op, 6 m	Cementless	GMED bilat.	Trendelenburg + partial BW	RMS %MVIC	Pre-op hyperactive operated GMED; at 6 m: operated ↓, contralateral ↑
[23]	11 THA + 11 ctrls	Static stance stabilizer activity	45–60 days	Anterior minimally invasive	GMED, GMAX, TFL, S	Bipodal + unipodal stance	RMS %MVIC	GMED/GMAX/TFL markedly ↑ vs. controls; early compensatory TFL strategy

Abbreviations: GMED = gluteus medius; GMAX = gluteus maximus; TFL = tensor fasciae latae; S = sartorius; LH = lateral hamstring; RF = rectus femoris; ES = erector spinae; BW = body weight; RMS = Root Mean Square; MVIC = Maximum Voluntary Isometric Contraction; ↑ = increase; ↓ = decrease.

## Data Availability

No new data were created or analyzed in this study. Data sharing is not applicable.

## Supplementary Materials

The following supporting information can be downloaded from https://www.mdpi.com/article/10.3390/jcm15010400/s1, Table S1: PRISMA checklist; Table S2: Search strategies.
