# Supplementary material for: Electromyography After Total Hip Arthroplasty: A Systematic Review of Neuromuscular Alterations and Functional Movement Patterns
Source: J Clin Med. 2026 Jan 5;15(1):400. doi: 10.3390/jcm15010400 (PMC12786741; doi:10.3390/jcm15010400)
Supplement: Supplementary file 1 [file jcm-15-00400-s001.zip › jcm-4058734-Table S1.pdf]

Table S1. Search strategies.

| Database | Search strategy |
|---|---|
| **PubMed/MEDLINE** | Searches were conducted using a combination of Medical Subject Headings (MeSH) and free-text terms related to *total hip arthroplasty* or *hip replacement* AND *electromyography* (including surface EMG, needle EMG, fine-wire EMG, and high-density EMG). Terms referring to functional tasks (e.g., gait, walking, stance, functional activities) were included where appropriate. No language or date restrictions were applied. |
| **Embase** | Database searches combined Emtree terms and free-text keywords related to *total hip arthroplasty* or *hip replacement* AND *electromyography*. Filters and indexing terms specific to Embase were applied to capture studies using surface, needle, fine-wire, and quantitative EMG techniques during dynamic or static motor tasks. |
| **Scopus** | Searches were performed using title, abstract, and keyword fields with combinations of terms related to *total hip arthroplasty* or *hip replacement* AND *electromyography* or *EMG*. Additional terms describing functional tasks (e.g., gait, walking, stance) were included to refine retrieval of relevant studies. |
| **Web of Science Core Collection** | Topic searches (TS) were conducted using combinations of terms related to *total hip arthroplasty* or *hip replacement* AND *electromyography* or *EMG*. No restrictions on publication year or language were applied. |
| **CINAHL** | Searches combined controlled vocabulary (CINAHL Headings) and free-text terms related to *total hip arthroplasty* AND *electromyography*, with additional keywords referring to movement analysis and functional motor tasks. |
| **IEEE Xplore** | Searches focused on free-text terms related to *total hip arthroplasty* or *hip replacement* AND *electromyography* or *EMG*, targeting engineering- and technology-oriented studies involving EMG signal acquisition, processing, or analysis in postoperative populations. |
| **Cochrane CENTRAL** | Searches were conducted using combinations of keywords related to *total hip arthroplasty* AND *electromyography* to identify controlled trials and prospective studies involving EMG assessment after THA. |
| **Google Scholar** | Searches were performed using combinations of the terms *total hip arthroplasty*, *hip replacement*, and *electromyography*. Results were sorted by relevance, and the first 200 records were screened, in line with common methodological recommendations. |
